# An Introductory Discourse on Medical Education, Delivered to the Students of Geneva Medical College, October 1, 1844

**Published:** 1845-01

**Authors:** 


*An Introductory Discourse on Medical Education, delivered to the Students of Geneva Medical College, October* 1, 1844. By CHARLES LEE, A. M., M. D., Professor of General Pathology and Materia Medica in Geneva College. Published by the Class. pp. 40.

This is a learned and able production. We are particularly pleased with it because of the importance the author attaches to a sound preparatory education for those who design to engage in the study of medicine. It is not likely that we shall be able, very soon, to raise the standard so high as he proposes, but it is well to keep an object of so much importance steadily and prominently in view.

In a future number we may find room for a few extracts, and perhaps may add an occasional remark on some of the points discussed.

